# Solubilization capacity of nonionic surfactant micelles exhibiting strong influence on export of intracellular pigments in *Monascus fermentation*

**DOI:** 10.1111/1751-7915.12039

**Published:** 2013-02-20

**Authors:** Biyu Kang, Xuehong Zhang, Zhenqiang Wu, Hanshi Qi, Zhilong Wang

**Affiliations:** 1 School of Biological Science and Engineering, South China University of TechnologyGuangzhou, 510006, China; 2 School of Pharmacy, Shanghai Jiao Tong UniversityShanghai, 200240, China; 3 State Key Laboratory of Microbial Metabolism, Shanghai Jiao Tong UniversityShanghai, 200240, China

## Abstract

In this study, perstractive fermentation of intracellular *Monascus* pigments in nonionic surfactant micelle aqueous solution had been studied. The permeability of cell membrane modified by nonionic surfactant might have influence on the rate of export of intracellular pigments into its extracellular broth while nearly no effect on the final extracellular pigment concentration. However, the solubilization of pigments in nonionic surfactant micelles strongly affected the final extracellular pigment concentration. The solubilization capacity of micelles depended on the kind of nonionic surfactant, the super-molecule assembly structure of nonionic surfactant in an aqueous solution, and the nonionic surfactant concentration. Elimination of pigment degradation by export of intracellular *Monascus* pigments and solubilizing them into nonionic surfactant micelles was also confirmed experimentally. Thus, nonionic surfactant micelle aqueous solution is potential for replacement of organic solvent for perstractive fermentation of intracellular product.

## Introduction

The current microbial industry of intracellular product fermentation usually follows a basic procedure, i.e. cultivation of microbes to achieve their maximum intracellular product concentration, followed by collection of the microbial cells, and finally separation of the intracellular product by destroy of the microbial cells. The limitation of this procedure is obvious. Product inhibition/degradation usually occurs at a high intracellular product concentration. At the same time, the downstream processing of intracellular product is very complex due to the cell disruption for extraction of intracellular product. Application of molecular engineering approaches for modifying outer membrane structure and expressing membrane-active peptides is carried out to improve the permeability of cell membrane (Chen, 2007; Idiris *et al*., 2010; Martin *et al*., 2010; Hou *et al*., 2012). Even cell surface display is developed to direct synthesis of microbial product on the outside of cell membrane (Sun *et al*., 2012). Chemical treatments of microbial cells, such as incubation in organic solvent (Kondo *et al*., 2000), nonionic surfactant micelle aqueous solution (Malik *et al*., 2012) etc., have also been applied to release intracellular product.

Recently, export of intracellular product by microbial fermentation in a water-organic solvent two-phase system, which is known as ‘milking processing’, has attracted the attention of bioprocessing engineers (Hejazi *et al*., 2004; Hejazi and Wijffels, 2004; Kleinegris *et al*., 2011a). The famous log P criterion indicates that only the organic solvent with a relatively higher log P value (log P is the partitioning coefficient of an organic compound in the water-octanol two-phase system) is biocompatible to microbes (Laane *et al*., 1987). However, most microbial products, especially the products with a moderate log P, have limited solubility in the high log P organic solvent (Meyer *et al*., 2006). It is found that the solubility of intracellular product in the organic solvent with a high log P is usually very low (Leon *et al*., 2003). When screening ‘milking’ solvent by cultivation of *Nannochloropsis* sp. in alkanes or alcohols, it was observed that the export of intracellular lipids increased significantly with the decrease of organic solvent log P while the corresponding biocompatibility decreased markedly (Zhang *et al*., 2011). Milking intracellular β-carotene has been realized by cultivation of the microalgae *Dunaliella salina* in the water-dodecane two-phase system (Hejazi *et al*., 2004). However, the rate of microalgae autolysis was nearly equal to that of microalgae growth in this process (Kleinegris *et al*., 2011b). Thus, permeability and biocompatibility become critical issues in selection of an organic solvent for ‘milking processing’.

Surfactant forms micelle pseudophase in an aqueous solution at the surfactant concentration above its critical micelle concentration (CMC). The micelle aqueous solution is a two-phase system where one is an aqueous solution and the other is a micelle pseudophase. A nonionic surfactant micelle aqueous solution as a special two-phase system has been used to extract the relatively higher polar product in extractive fermentation, such as l-phenylacetylcarbinol (Xue *et al*., 2010), 1-butanol (Dhamole *et al*., 2012) etc. Especially, a nonionic surfactant micelle aqueous solution above a certain temperature (cloud point) separates into a surfactant dilute phase and a coacervate phase (surfactant-rich phase), which is known as cloud point system. The cloud point system is extensively studied for extraction of metal ions, organic compounds and biomaterials (Hinze and Pramauro, 1993; Ingram *et al*., 2012) and is also developed as a novel two-phase system for extractive fermentation (Wang *et al*., 2004; Wang and Dai, 2011). *Monascus* pigments, which include red pigments, orange pigments and yellow pigments, are intracellular products of microbial fermentation (Juzlova *et al*., 1996). In the previous work, submerged cultivation of *Monascus* sp. in the nonionic surfactant Triton X-100 micelle aqueous solution ‘milked’ the intracellular pigments into the extracellular broth successfully, in which maintenance of microbial growth and export of intracellular pigments were fulfilled at the same time. The solubilization of pigments in the nonionic surfactant micelles was also confirmed (Hu *et al*., 2012a). The milking processing, which is actual perstractive fermentation, involves the secretion of intracellular product across cell membrane to extracellular broth and then extraction of the extracellular product into nonionic surfactant micelles. High extracellular pigment concentration was also achieved by perstractive fermentation using two-stage operation mode, i.e. cultivation of microbes in an aqueous solution for microbial growth in the first stage and then perstractive fermentation in a nonionic surfactant micelle aqueous solution for production of extracellular pigments in the second stage (Hu *et al*., 2012b).

The passive diffusion of small hydrophobic organic product across cell membrane is described as (Koley and Bard, 2010)


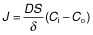
(1)

where *J* is the flux of intracellular product across cell membrane; *σ* is the thickness of cell membrane; *D* is the diffusion coefficient of product in the cell membrane; *S* is the solubility of intracellular product in the cell membrane; *C*_i_ is the intracellular product concentration; and *C*_o_ is the extracellular product concentration. This equation means the rate of export of intracellular product across cell membrane depends on the cell membrane permeability, which includes the character of cell membrane structure (*σ*, *D*, *S*) and the intracellular product itself (*D*, *S*), and the intracellular and extracellular product concentration difference (*C*_i_ − *C*_o_). When the extracellular product concentration reaches to the intracellular product concentration, the diffusion of intracellular product into its extracellular broth is ceased. Solubilization of pigments in the nonionic surfactant micelles decreases the pigment concentration in the extracellular aqueous phase (*C*_o_), which increases the intracellular and extracellular pigment concentration difference and then intensifies the export of intracellular pigments in the perstractive fermentation of *Monascus* pigment process. On the other hand, cell membrane lipid may also be solubilized by the nonionic surfactant micelle solution, which modifies the structure and then the permeability of cell membrane (Koley and Bard, 2010). Whether the modification of cell membrane by nonionic surfactant involving the intensification of intracellular pigment export in the perstractive fermentation process or not remains unclear. At the same time, high extracellular *Monascus* pigment concentration was achieved by two-stage perstractive fermentation in a nonionic surfactant micelle aqueous solution, which was attributed to the elimination of product inhibition and/or degradation. However, the product inhibition or degradation did not determine experimentally (Hu *et al*., 2012b). The mechanism concerning the elimination of product inhibition/degradation should be further studied.

Perstractive fermentation of *Monascus* pigments using two-stage operation mode was set up as a model in the present work. The biocompatibility and secretion of intracellular pigments by different polymers/nonionic surfactants were screened. The effect of the state of microbial cells, the super-molecule assembly structure of nonionic surfactant in an aqueous solution and the nonionic surfactant concentration on releasing intracellular product was examined. The stabilization effect of nonionic surfactant on product degradation was further determined by perstractive fermentation of intracellular *Monascus* pigments in a nonionic surfactant micelle aqueous solution.

## Results

### Effect of nonionic surfactants on *Monascus anka*

**Figure 1:**
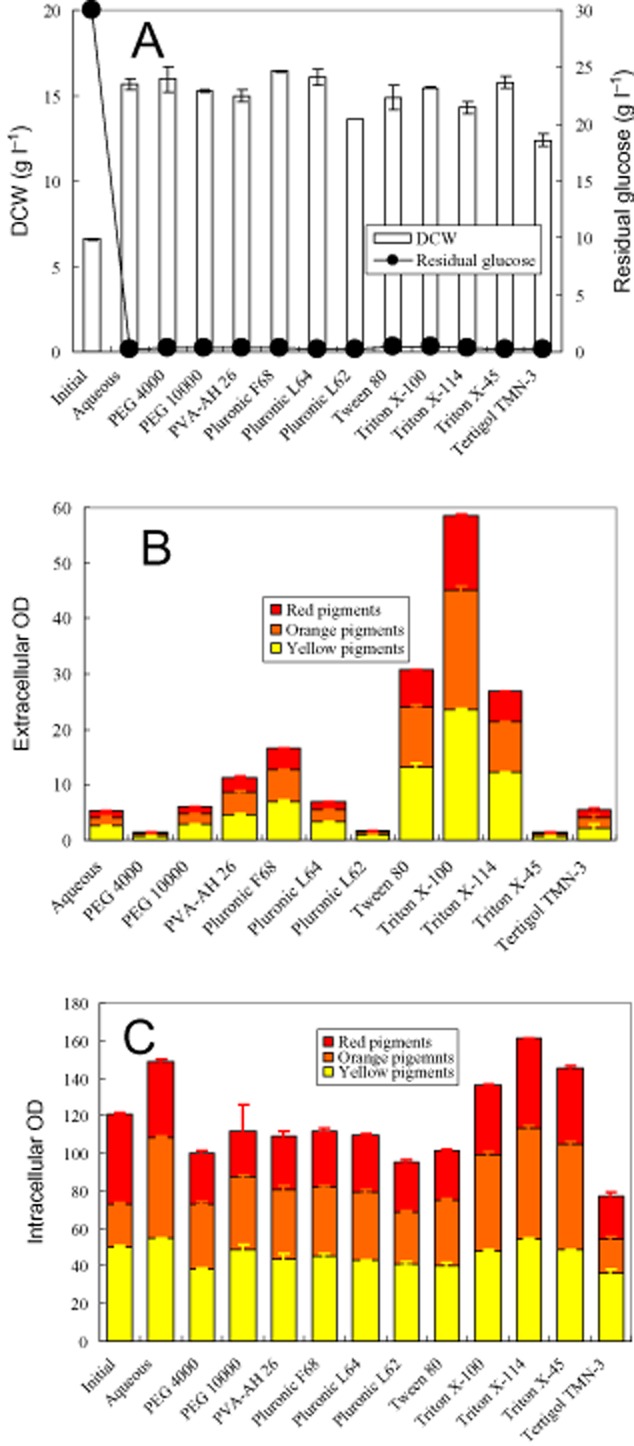
Screening nonionic surfactant by the second-stage cultivation of *Monascus anka*. (A) Residual glucose and biomass; (B) extracellular pigment concentration; (C) intracellular pigment concentration.

Perstractive fermentation using two-stage operation mode was carried out. The second-stage perstractive fermentation in different polymer/nonionic surfactant micelle aqueous solutions was determined as shown in Fig 1. The basic information about these polymers/nonionic surfactants was listed in Table 1. At the initial, approximately 7.2 g l^−1^ DCW (dry cell weight), corresponding to total intracellular red, orange and yellow pigments approximately 120 OD, and 30 g l^−1^ glucose were loaded. Utilizing the second-stage fermentation in the aqueous medium (absence of surfactant/polymer) as control, the effect of polymers/nonionic surfactants on *Monascus* growth and consumption of glucose was examined (Fig. 1A). The result indicated that *Monascus anka* grew well in these polymer/nonionic surfactant aqueous media. In the control, the increase of biomass only led to limited increase of intracellular pigment concentration. The extracellular pigment concentration was very low, too. In the presence of polymer/nonionic surfactant in the aqueous medium, increase or decrease of intracellular pigment concentration was observed in a few cases (Fig. 1C). However, high extracellular pigment concentration in the nonionic surfactant Triton X-100, Triton X-114 and Tween 80 micelle aqueous solution was found (Fig. 1B). These results indicated that releasing intracellular pigments depended on the kind of polymers/nonionic surfactants despite that *Monascus* sp. grew very well in all examined polymers/nonionic surfactant aqueous solutions.

**Table 1:** Basic information of screening polymers/nonionic surfactants

Polymer/surfactant	Hydrophobic group	Oxyethylene unit	HLB^a^	Cloud point (^o^C)	CMC^b^ (mM)
Tergitol TMN-3		3	8.1	Insoluble	
Triton X-45	*t*-Octylphenoxy	4.5	9.8	Dispersible	0.103
Triton X-114	*t*-Octylphenoxy	7.5	12.3	25	0.2
Triton X-100	*t*-Octylphenoxy	9.5	13.4	66	0.21
Tween 80	Sorbitol monooleate	20		> 100	0.012
Pluronic L 62	Polyoxypropylene	Polyoxyethylene	7	Double cloud point	
Pluronic L 64	Polyoxypropylene	Polyoxyethylene	7–14	> 60	
Pluronic F 68	Polyoxypropylene	Polyoxyethylene	> 24	> 100	
PVA-AH 26		Polyvinylalcohol		> 100	
PEG 10000		Polyoxyethylene		> 100	
PEG 4000		Polyoxyethylene		> 100	

a Hydrophile–lipophile balance.

b Critical micelle concentration.

### Releasing intracellular pigments in Triton X-100 micelle solution

**Figure 2:**
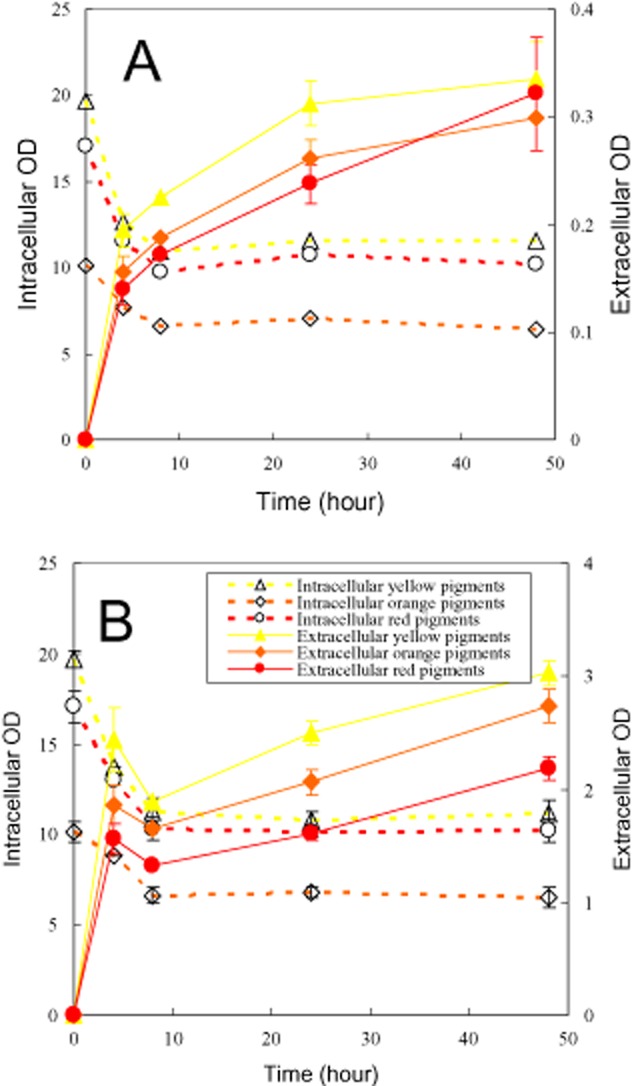
Releasing intracellular pigments (mycelia collected from the first-stage fermentation). (A) Aqueous solution; (B) Triton X-100 micelle aqueous solution.

**Figure 3:**
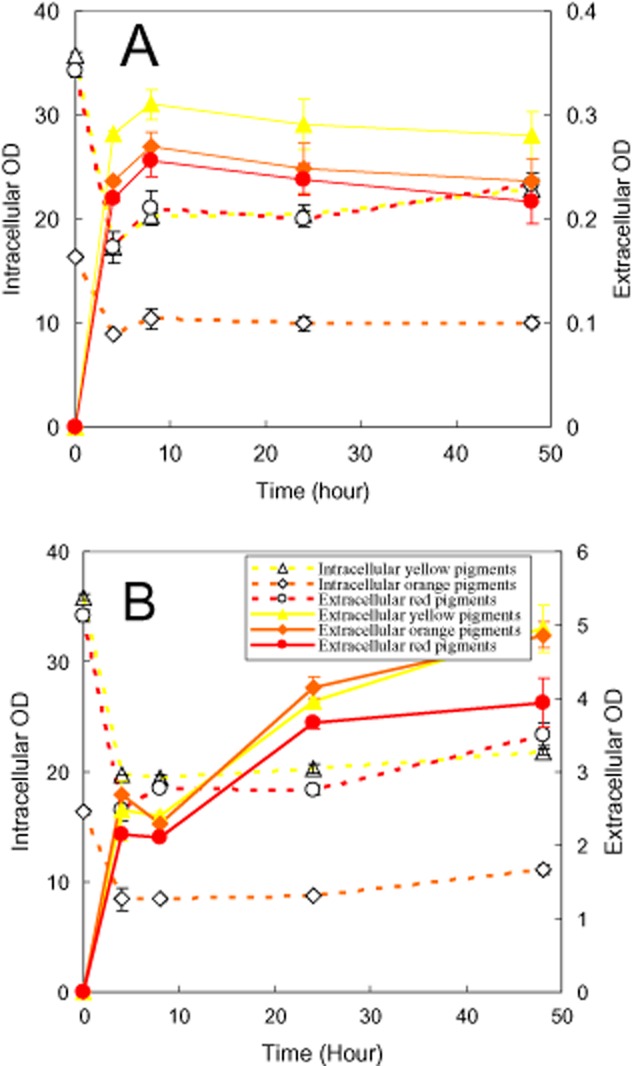
Releasing intracellular pigments (mycelia collected from the second-stage perstractive fermentation). (A) Aqueous solution; (B) Triton X-100 micelle aqueous solution.

**Figure 4:**
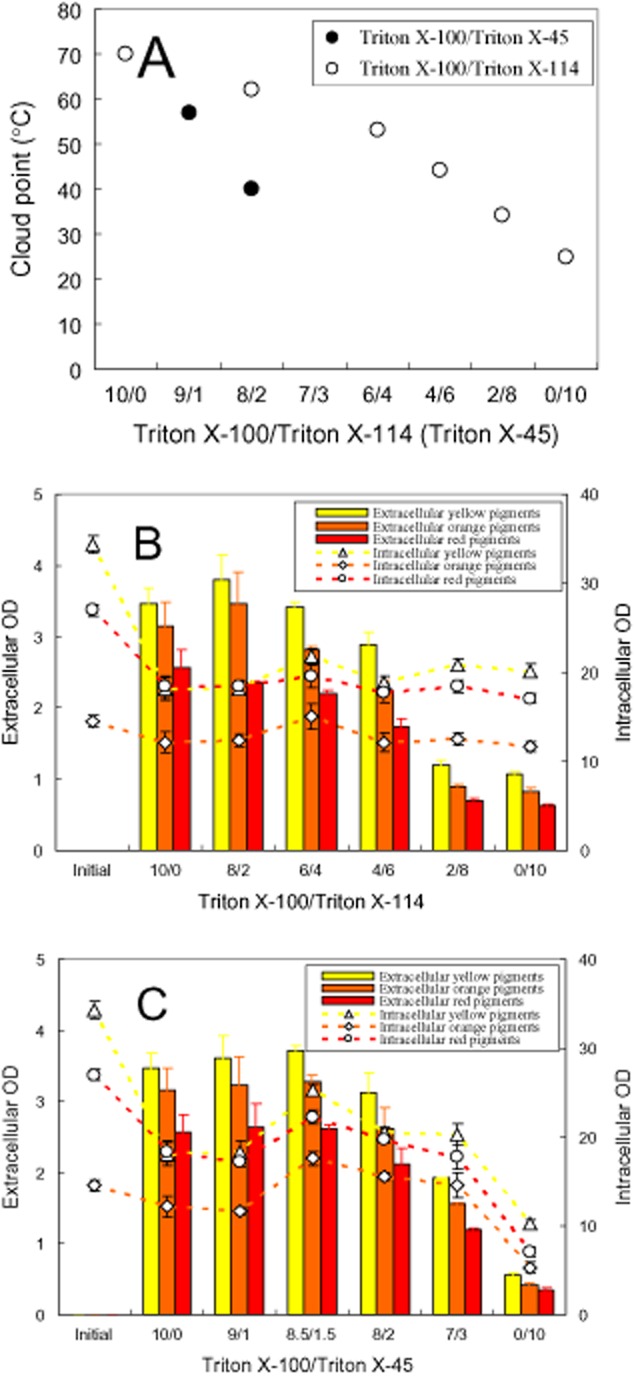
Effect ratio of Triton X-100/Triton X-114 (Triton X-45) on releasing intracellular pigments. (A) Cloud point; (B) Triton X-100/Triton X-114; (C) Triton X-100/Triton X-45.

**Figure 5:**
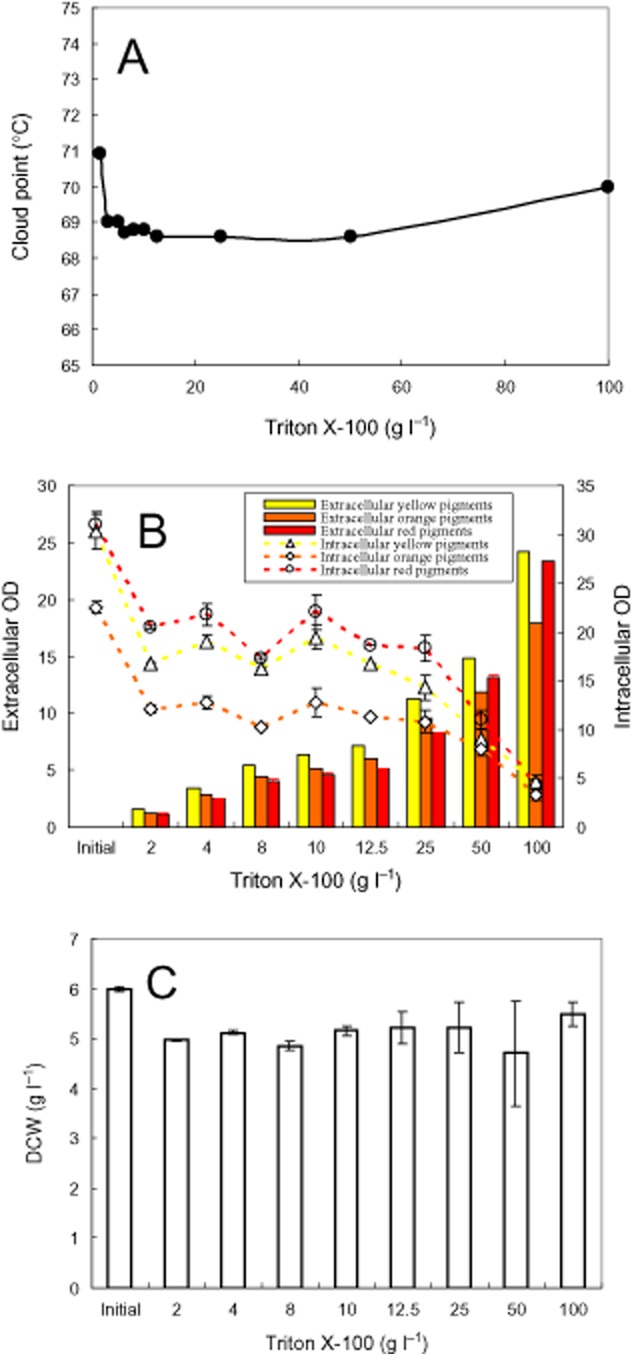
Effect of Triton X-100 concentration on releasing intracellular pigments. (A) Cloud point; (B) intracellular and extracellular pigment concentration; (C) DCW.

Triton X-100 exhibited excellent ability to export of intracellular pigments, in which production of pigments occurred due to the microbial growth (Fig. 1). In order to study the releasing intracellular pigments, microbial growth and pigment formation were precluded by incubation of mycelia in the aqueous solution without carbon and nitrogen source. Mycelia collected from the first-stage fermentation were used to study the time-course of releasing intracellular pigments (Fig. 2). The release of intracellular pigments in the aqueous solution without Triton X-100 was used as control (Fig. 2A). Rapid export of intracellular pigments occurred during the first 4 h then the rate of releasing intracellular pigments slowed down. The profile of yellow (410 nm), orange (470 nm) and red (510 nm) pigment absorbance in both intracellular and extracellular were very similar, which hinted the similar spectrum of intracellular and extracellular *Monascus* pigments. Compared with the control, the presence of Triton X-100 exhibited a similar trend except that the corresponding extracellular pigment concentration had increased nearly 10 times (Fig. 2B). It must be pointed out that the decrease of intracellular pigment concentration was much higher than the increase of extracellular pigment concentration during the first 4 h. The similar phenomenon was also observed in the following experiments (Figs 3–5). All of these indicated pigment degradation had occurred.

According to Eq. (1), the export of intracellular pigments relates not only to the pigment concentration difference across the cell membrane but also to the permeabilization of cell membrane. The effect of Triton X-100 on the permeability of cell membrane was checked by releasing intracellular pigments, in which mycelia were collected from the second-stage perstractive fermentation in Triton X-100 micelle aqueous solution (Fig. 3). Different from mycelia collected from the first-stage fermentation in the aqueous solution (Fig. 2), the possible effect of Triton X-100 on the structure of cell membrane occurred in the second-stage perstractive fermentation in Triton X-100 aqueous solution. Compared with Fig. 2A, the rate of releasing intracellular pigments was enhanced, where the extracellular pigment absorbance reached to the highest value at the eighth hour (Fig. 3A). However, the highest absorbance was nearly the same as that of Fig. 2A. These results indicated that the growth of mycelia in Triton X-100 micelle aqueous medium might affect the cell membrane permeability and then the rate of release of intracellular pigments while had nearly no effect on the final extracellular pigment concentration. On the other hand, the extracellular pigment concentration increased markedly from the aqueous solution (Fig. 3A) to the Triton X-100 micelle aqueous solution (Fig. 3B), which indicated that the nonionic surfactant strongly influence on the extracellular pigment concentration. Furthermore, mycelia from the first-stage fermentation (Fig. 2B) and the second-stage perstractive fermentation in the Triton X-100 micelle aqueous medium (Fig. 3B) also exhibited as a similar trend of changing extracellular pigment concentration, in which the high intracellular pigment concentration increased the extracellular pigment concentration in the Triton X-100 micelle aqueous solution (Fig. 3B versus Fig. 2B).

### Effect of mixture nonionic surfactants

Triton X-100, Triton X-114 and Triton X-45 belong to the same series of nonionic surfactants with the same hydrophobic moiety while the hydrophilic moiety differs in numbers of oxyethylene unit. The super-molecule structures of Triton X-100, Triton X-114 and Triton X-45 in an aqueous solution at room temperature are micelles, cloudy and dispersible respectively (Table 1). The different effect of these nonionic surfactants on extracellular pigment concentrations (Fig. 1B) spurs us to examine the mixture nonionic surfactant effect on releasing intracellular pigments. The cloud point of Triton X-100/Triton X-114 and Triton X-100/Triton X-45 mixture was determined as shown in Fig 4A. The cloud point of Triton X-100/Triton X-114 mixture was between the cloud point of Triton X-100 and that of Triton X-114. Triton X-100 and Triton X-45 mixture were dispersible at the ratio of Triton X-100 to Triton X-45 between 7:3 and 0:10. Only at very high ratio of Triton X-100 to Triton X-45, the nonionic surfactant mixture formed micelles in the aqueous solution and exhibited cloud point. The initial state of Fig. 4B and C represented the intracellular pigment concentration of the loaded mycelia. In the presence of Triton X-100/Triton X-114 mixture, the intracellular pigment concentration remained nearly unchangeable while the extracellular pigment concentration decreased markedly with the ratio of Triton X-100 to Triton X-114 changing from 4:6 to 0:10 (Fig. 4B). It indicated that substantial pigment degradation also occurred at high ratio of Triton X-100 to Triton X-114. The effect of Triton X-100/Trion X-45 mixture exhibited a similar trend to that of Triton X-100/Triton X-114 mixture. However, the mixtures were dispersible in the aqueous solution at the ratio of Triton X-100 to Triton X-45 below 7:3, in which decrease of both intracellular and extracellular pigment concentration was observed. This result was consistent with the low intracellular and extracellular pigment concentration of the second-stage perstractive fermentation in Triton X-45 aqueous solution (Fig. 1).

### Effect of nonionic surfactant concentration

The effect of Triton X-100 concentration on cloud point was determined (Fig. 5A). The cloud point decreased to the lowest temperature and then increased with the increase of Triton X-100 concentration. The effect of Triton X-100 concentration on releasing intracellular pigments was examined (Fig. 5B). The extracellular pigment concentration increased while the intracellular pigment concentration decreased with the increase of Triton X-100 concentration. Interestingly, the pigment degradation disappeared with the increase of Triton X-100 concentration, which exhibited as the absorbance of intracellular and extracellular pigments in the micelle solution with 100 g l^−1^ Triton X-100 was nearly the same as that of initial intracellular pigment concentration. After releasing intracellular pigments with different concentration of Triton X-100 micelle aqueous solution, DCW was also determined as shown in Fig. 5C. In comparison with the initial DCW, substantial decrease of DCW was also found after treatment with Triton X-100 micelle aqueous solution. However, the effect of Triton X-100 concentration on decrease of DCW submerged into the errors of DCW analysis.

### Time-course of the second-stage perstractive fermentation

**Figure 6:**
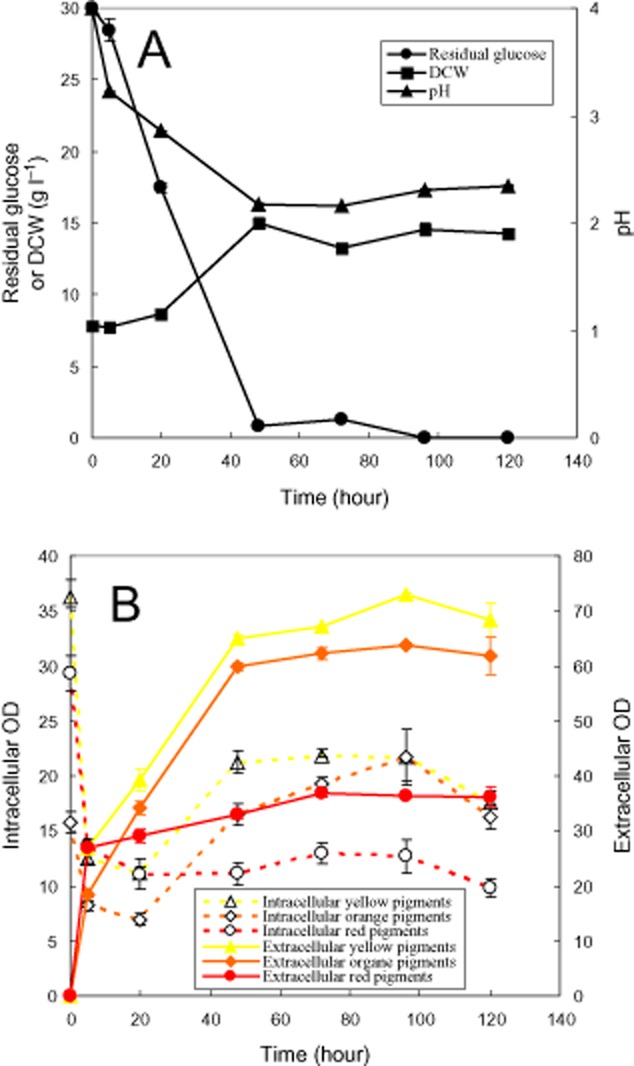
Time-course of the second-stage perstractive fermentation. (A) Residual glucose, pH and biomass; (B) intracellular and extracellular pigments.

Due to the strong elimination of pigment degradation in the micelle aqueous solution with high Triton X-100 concentration (Fig. 5B), the second-stage perstractive fermentation in the aqueous medium with 100 g l^−1^ Triton X-100 was carried out as shown in Fig 6. The operation temperature was much below the cloud point of the Triton X-100 aqueous solution (Fig. 5A). The increase of viscosity by the presence of high nonionic surfactant concentration was low enough to keep the aqueous medium vigorous shaken in the fermentation process. The increase of DCW, glucose consumption and pH change [corresponding to consumption of (NH_4_)_2_SO_4_] indicated that mycelia grew very well in the second-stage perstractive fermentation (Fig. 6A). Based on the changing absorbance of intracellular and extracellular pigments, the time-course of the second-stage perstractive fermentation could be divided into four phases (Fig. 6B): from the beginning to the fourth hour was the first phase, where the rate of pigment production was slower than that of releasing intracellular pigments. The intracellular pigment concentration decreased while the extracellular pigment concentration increased rapidly. From the 4th hour to the 20th hour was the second phase, where the rate of pigment production was nearly equal to the rate of releasing intracellular pigments. The intracellular pigment concentration kept nearly a constant while the extracellular pigment concentration increased rapidly. From the 20th hour to the 48th hour was the third phase, where the rate of pigment production was faster than that of releasing intracellular pigments. It might be attributed to the micelle solubilization capacity was nearly saturated at the high extracellular pigment concentration. Both intracellular and extracellular pigment concentration was increased. Before the 48th hour, glucose consumption, pH decrease and the microbial growth had nearly completed (Fig. 6A). Then after, the microbial fermentation came into the fourth phase, where all the parameters, such as DCW, pH, intracellular and extracellular concentration, came to nearly a constant except a few decrease of intracellular pigment concentration. It should be pointed out that yellow pigments and red pigments were the main components of intracellular pigments at the initial state while yellow pigments and orange pigments became the main components at the end of perstractive fermentation, which might be attributed to the changing pH during the second-stage perstractive fermentation (Fig. 6A).

## Discussion

The effect of nonionic surfactant on export of intracellular product may be involved modification of cell membrane structure and then adjustment of *D* (diffusion coefficient of product in the cell membrane) and *S* (solubility of intracellular product in the cell membrane) as shown in Eq. (1). Utilization of mycelia collected from the first-stage fermentation as control (Fig. 2), the mycelia collected from the second-stage perstractive fermentation, where mycelia had been incubated in the Triton X-100 micelle aqueous medium for 3 days, was used to study the effect of nonionic surfactant on the permeability of cell membrane (Fig. 3). The mycelia collected from the second-stage perstractive fermentation exhibited a rapid release of intracellular pigments in the aqueous solution (Fig. 3A versus Fig. 2A), which indicated that the incubation of mycelia in the Triton X-100 aqueous medium might modify the cell membrane structure and then adjust the permeability of cell membrane. This result is consistent with the effect of nonionic surfactant Triton X-100 on membrane permeability of a single HeLa cell (Koley and Bard, 2010). However, these two kinds of mycelia exhibited near the same extracellular pigment concentration even the mycelia collected from the second-stage perstractive fermentation maintained a relatively higher intracellular pigment concentration (Figs 2A and 3A). No substantial difference was also observed by releasing intracellular pigments of these two kinds of mycelia in the Triton X-100 micelle aqueous solution (Figs 2B and 3B). Both of them indicated that the modification of cell membrane permeability had nearly no effect on final extracellular pigment concentration.

Solubilization of pigments in nonionic surfactant micelles (Wang *et al*., 2003; Mehling *et al*., 2012) decreases the pigment concentration in the aqueous solution and then increases the intracellular and extracellular pigment concentration difference. According to Eq. (1), the decrease of intracellular pigment concentration in the aqueous solution (*C*_o_) by solubilization not only enhances the rate of export of intracellular pigments but also increases the final extracellular pigment concentration (sum of pigments solubilization in micelles and in the aqueous solution). Such as the presence of nonionic surfactant micelles in an aqueous solution increased the extracellular pigment concentration (Figs 1–3). The enhancement of releasing intracellular pigments was also intensified by increase of nonionic surfactant concentration (Fig. 5B). On the other hand, high intracellular pigment concentration also led to the increase of extracellular pigment concentration (Fig. 2B versus Fig. 3B). Based on the similar principle, addition of polymeric resin to intensify exporting intracellular pigments is also reported in *Monascus* fermentation (Evanst and Wang, 1984). However, the enhancement of exporting intracellular pigments in the presence of nonionic surfactant is related to the super-molecule assembly structure of nonionic surfactant in an aqueous solution. Super-molecule assembly structure of Triton X-100, Triton X-114 and Triton X-45 in an aqueous solution is micelles, cloudy and dispersible respectively (Wang *et al*., 2008). The non-micelle structure of Triton X-45 in an aqueous solution led to the lowest extracellular pigment concentration (Fig. 4C). Similar result was also observed for series of Pluronic L62 (dispersible), Pluronic L64 (micelle), Pluronic F68 (micelle) (Wang and Feng, 2010) as shown in Fig. 1, where the lowest extracellular pigment concentration was also found in the dispersible Pluronic L62 aqueous medium. Triton X-114 as well as the mixtures with low ratio of Triton X-100 to Trion X-114 formed cloud point system (Fig. 4A). A relatively lower extracellular pigment concentration was also observed (Fig. 4B). However, the extracellular pigment concentration was still higher than that of releasing intracellular pigments in the aqueous solution (Fig. 2A). Although solubilization of organic compound in the coacervate phase of a cloud point system has been confirmed experimentally, comparison of the solubilization capacity between the coacervate phase and micelles is still unclear (Sakulwongyai *et al*., 2000; Wang *et al*., 2003). All of these indicate that the export of intracellular pigments is related to the solubilization capacity of the super-molecule assembly structures of nonionic surfactant in the aqueous solution.

A rapid degradation of pigments during the first 4 h was observed in the releasing intracellular pigment (Figs 2–5). It is reported that one unit absorbance of *Monascus* pigments at 480 nm corresponds to pigment weight 15 mg l^−1^ (Hajjaj *et al*., 2012). Accordingly, the pigment degradation as shown in Fig. 5B was less than 0.5 g l^−1^. A substantial decrease of DCW after treatment with Triton X-100 micelle aqueous solution in comparison with the initial DCW (Fig. 5C) hints other cell component degradation or secretion into extracellular broth also occurs. *Monascus* pigments are moderate polar compounds. The log P of red pigments, orange pigments, and yellow pigment is approximately 2, 2.7 and 3.1 respectively (Jung *et al*., 2003). The solubilization of these moderate polar pigments in nonionic surfactant micelles prevents them from direct contact with microbial cells. The indirect contact between microbial cells and the pigments solubilized in micelles decreases the rate of pigment degradation by the microbial cells (Dai *et al*., 2010; Wang, 2011). It exhibited as the pigment degradation disappeared with the increase of nonionic surfactant concentration (Fig. 5B). Eliminating pigment degradation leading to high extracellular pigment concentration was also achieved by perstractive fermentation in nonionic surfactant micelle aqueous medium (Fig. 6B) while the increase of intracellular and extracellular pigment concentration was very limited in the aqueous medium (Fig. 1B and C).

Extractive fermentation of extracellular product has been studied extensively using water-organic solvent two-phase system (Daugulis, 2001; Malinowski, 2001; Fernandes *et al*., 2003; Straathof, 2003; Heipieper *et al*., 2007), aqueous two-phase system (Kumn, 1980), cloud point system (Wang and Dai, 2011) and ionic liquids (Cull *et al*., 2000). The advantage of extractive fermentation mainly includes elimination of substrate/product inhibition, enhancement of the solubility of hydrophobic substrate, prevention of product from further degradation, and partial purification of product by the extraction process. Most organic solvents with high log P are poor solvent for extraction of moderate polar organic compounds (Meyer *et al*., 2006). However, extractive fermentation has to carry out in hydrophobic organic solvent-water two-phase system to achieve high biocompatibility (Laane *et al*., 1987). Thus, only limited concentration of extracellular lipids is observed by perstractive fermentation of microalgae intracellular lipid in a water-organic solvent two-phase system (Zhang *et al*., 2011). On the contrary, cultivation of microalgae in a cloud point system for milking intracellular lipid is reported recently (Glembin *et al*., 2013). The growth of *Monascus* in the different polymer/nonionic surfactant aqueous media (Figs 1A and 6A) means good biocompatibility is maintained by perstractive fermentation in a nonionic surfactant micelle aqueous solution. The good biocompatibility and strong solubilization capacity make nonionic surfactant micelle aqueous solution potential for replacement of water-organic solvent two-phase system for export of intracellular product in fermentation process.

## Experimental procedures

### Polymers and nonionic surfactants

Polyethylene glycerol polymers, including PEG 4000 and PEG 10000 (Shanghai Chemical Agent, China); Polyvinylalcohol PVA-AH 26 (Shanghai Chemical Agent); Triblock copolymers, including Pluronic L62, Pluronic L64 (Zhejiang Huangma Chemical, Zhejiang, China) and Pluronic F68 (Fluka); and nonionic surfactants, including Tween 80 (Shanghai Chemical Agent), Triton X-100, Triton X-114, Triton X-45 and Tergitol TMN-3 (Fluka), were used without further purification. These polymers/nonionic surfactants were screened as potential agents for the second-stage perstractive fermentation as described in Fig. 1. The basic information of polymers/nonionic surfactants was listed in Table 1.

### Determination of cloud point

A series of mixture nonionic surfactant (4 g l^−1^) aqueous solution with different ratio of Triton X-100 to Triton X-114 (Triton X-45) (Fig. 4) or series of Triton X-100 aqueous solution with surfactant concentration ranging from 2.5 to 100 g l^−1^ (Fig. 5) were prepared with distilled water. The nonionic surfactant aqueous solution samples (10 ml) were put into 25 ml glass tubes and incubated in a thermostat water bath. The samples were heated with a temperature step of 1°C and held at each temperature for 2 min to reach thermo-equilibrium. The transition of nonionic surfactant aqueous solution from clear to cloudy was determined visually and the corresponding temperature was denoted as cloud point.

### Microorganisms and cultivation

*Monascus anka* (China Center of Industrial Culture Collection, CICC 5013) was maintained on potato dextrose agar (PDA) medium (potato dextrose 200 g, glucose 20 g and agar 15–20 g per litre of water) and preserved at 4°C.

Seed culture medium consisted of glucose 20 g, (NH_4_)_2_SO_4_ 4 g, peptone 10 g, KCl 0.5 g, KH_2_PO_4_ 4 g and FeSO_4_·7H_2_O 0.01 g per litre of tap water. The seed cultivation was carried out in a 100 ml Erlenmeyer flask with 25 ml of the seed culture medium. The flask was incubated at 30°C and shaken at 180 r.p.m. for 2 days.

Basic aqueous fermentation medium consisted of glucose 30 g, (NH_4_)_2_SO_4_ 3 g, KH_2_PO_4_ 5 g, CaCl_2_ 0.1 g and FeSO_4_·7H_2_O 0.01 g per litre of tap water. The initial pH was adjusted to 4 with 10% (v/v) hydrochloric acid before fermentation cultivation.

### Two-stage perstractive fermentation

Two-stage operation mode followed the same procedure as the previous work (Hu *et al*., 2012b). The basic operation was briefly described as following: the first-stage fermentation was carried out in the basic aqueous fermentation medium. Twenty-five millilitres of the aqueous medium was added in every 100 ml Erlenmeyer flask. After the seed cultivation, 2 ml of seed culture broth was withdrawn and added into the flasks, where the seed broth and the aqueous medium were combined. The flasks were incubated in 30°C shaken at 180 r.p.m. for 4 days. Two flasks as biological duplicates were used for independent analysis of intracellular pigment concentration and biomass.

After the first stage of microbial cultivation, the fermentation broth was subjected to centrifugation at 5000 r.p.m. for 10 min (Anke TDL 80-LB, China). The mycelia were collected on filter paper. Approximately 1.4 g of wet mycelia were resuspended in 25 ml of the aqueous medium in the presence of specified nonionic surfactant/polymer (8 g l^−1^) for screening experiment (Fig. 1). The cell resuspension solution was added to 100 ml Erlenmeyer flask, which was incubated in 30°C and shaken at 180 r.p.m. for 3 days. For the time-course of the second-stage perstractive fermentation experiment (Fig. 6), 100 g l^−1^ Triton X-100 was added. Two flasks as biological duplicates were used for independent analysis of pigment concentration, biomass and residual glucose concentration.

### Releasing intracellular pigments

An aqueous solution with the same components of the basic aqueous fermentation medium but deleted of carbon and nitrogen source was prepared. The free of carbon and nitrogen in this aqueous solution precludes the mycelia growth and pigment formation. After the first-stage cultivation of *Monascus* in the aqueous medium for 4 days (Figs 2, 4 and 5) or the second-stage perstractive fermentation in Triton X-100 micelle aqueous solution for 3 days (Fig. 3), the fermentation broth was subjected to centrifugation at 5000 r.p.m. for 10 min and the mycelia were collected. Wet mycelia (1 g) were resuspended into 25 ml of the aqueous solution or the aqueous solution in the presence of 4 g l^−1^ Triton X-100 (Figs 2 and 3), 4 g l^−1^ mixture nonionic surfactant Triton X-100/Triton X-114 (Triton X-45) (Fig. 4), or different concentration of Trion X-100 (Fig. 5), which was incubated and shaken under the same condition as the second-stage perstractive fermentation. The intracellular and extracellular pigment concentration was determined to check the release of intracellular pigments.

### Analysis methods

Fermentation broth was centrifuged at 5000 r.p.m. for 10 min. In some samples, such as liquid broth with low ratio of Triton X-100 to Triton X-45 or Triton X-114 is heterogeneous (Fig. 4), the samples were cooled at 4°C to form clear solution before centrifugation. One millilitre of the supernatant was directly diluted to determine the residual glucose concentration. The residual glucose in the fermentation broth was determined with a spectrophotometer by the standard 3, 5-dinitrosalicylic acid (DNS) method (Miller, 1959).

The mass concentration of extracellular pigments is difficult to determine due to the complex components in *Monascus* pigments. The concentration of *Monascus* pigments is usually estimated by measuring the corresponding absorbance (Babitha *et al*., 2007). Even pigment absorbance is also applied to represent the pigments concentration in the food additives *Monascus* colour by the standardization administration of the people's republic of China (GB 15961-2005). In the present work, One millilitre of the supernatant after centrifugation was properly diluted with 70% (v/v) ethanol aqueous solution. The absorbance of yellow, orange and red pigments in the corresponding ethanol aqueous solution was determined at a specific wavelength 410, 470 and 510 nm respectively. Pigment concentration was represented by the corresponding OD unit (multiplication of the absorbance with the sample dilution ratio).

After centrifugation, mycelia were collected for intracellular pigment concentration analysis. The mycelia were soaked in 70% (v/v) ethanol aqueous solution for 1 h, in which the ethanol aqueous solution was kept at the same volume as the original fermentation broth. The corresponding ethanol aqueous solution was subjected to intracellular pigment concentration analysis. Thus, the analysis of intracellular and extracellular pigment concentration were based on the same volume and the same solvent system of ethanol aqueous solution, which was convenient for comparing the pigment partitioning between the intracellular and the extracellular broth.

The mycelia in the ethanol solution were re-collected by centrifugation at 5000 r.p.m. for 10 min. DCW was estimated by keeping the mycelia at 50°C overnight to ensure the weight of mycelia did not change.

## Conflict of interest

None declared.
